# Effects of walking speeds and durations on the plantar pressure gradient and pressure gradient angle

**DOI:** 10.1186/s12891-022-05771-2

**Published:** 2022-08-30

**Authors:** Chi-Wen Lung, Pu-Chun Mo, Chunmei Cao, Keying Zhang, Fu-Lien Wu, Ben-Yi Liau, Yih-Kuen Jan

**Affiliations:** 1 Rehabilitation Engineering Lab, Department of Kinesiology and Community Health, University of Illinois at Urbana-Champaign, Champaign, IL USA; 2 Department of Creative Product Design, Asia University, Taichung, Taiwan; 3 Division of Sports Science and Physical Education, Tsinghua University, Beijing, China; 4 Department of Biomedical Engineering, Hungkuang University, Taichung, Taiwan

**Keywords:** Diabetic foot ulcers, Peak plantar pressure, Peak pressure gradient, Pressure gradient angle, Walking durations, Walking speeds

## Abstract

**Background:**

Walking exercise has been demonstrated to improve health in people with diabetes. However, it is largely unknown the influences of various walking intensities such as walking speeds and durations on dynamic plantar pressure distributions in non-diabetics and diabetics. Traditional methods ignoring time-series changes of plantar pressure patterns may not fully capture the effect of walking intensities on plantar tissues. The purpose of this study was to investigate the effect of various walking intensities on the dynamic plantar pressure distributions. In this study, we introduced the peak pressure gradient (PPG) and its dynamic patterns defined as the pressure gradient angle (PGA) to quantify dynamic changes of plantar pressure distributions during walking at various intensities.

**Methods:**

Twelve healthy participants (5 males and 7 females) were recruited in this study. The demographic data were: age, 27.1 ± 5.8 years; height, 1.7 ± 0.1 m; and weight, 63.5 ± 13.5 kg (mean ± standard deviation). An insole plantar pressure measurement system was used to measure plantar pressures during walking at three walking speeds (slow walking 1.8 mph, brisk walking 3.6 mph, and slow running 5.4 mph) for two durations (10 and 20 min). The gradient at a location is defined as the unique vector field in the two-dimensional Cartesian coordinate system with a Euclidean metric. PGA was calculated by quantifying the directional variation of the instantaneous peak gradient vector during stance phase of walking. PPG and PGA were calculated in the plantar regions of the first toe, first metatarsal head, second metatarsal head, and heel at higher risk for foot ulcers. Two-way ANOVA with Fisher’s post-hoc analysis was used to examine the speed and duration factors on PPG and PGA.

**Results:**

The results showed that the walking speeds significantly affect PPG (*P* < 0.05) and PGA (*P* < 0.05), and the walking durations does not. No interaction between the walking duration and speed was observed. PPG in the first toe region after 5.4 mph for either 10 or 20 min was significantly higher than 1.8 mph. Meanwhile, after 3.6 mph for 20 min, PPG in the heel region was significantly higher than 1.8 mph. Results also indicate that PGA in the forefoot region after 3.6 mph for 20 min was significantly narrower than 1.8 mph.

**Conclusions:**

Our findings indicate that people may walk at a slow speed at 1.8 mph for reducing PPG and preventing PGA concentrated over a small area compared to brisk walking at 3.6 mph and slow running at 5.4 mph.

## Introduction

Diabetes mellitus (DM) is a common metabolic disease due to abnormal insulin secretion or insulin action [1]. The global costs of DM are rapidly growing and are estimated to increase from $1.3 trillion in 2015 (global GDP 1.8%) to $2.2 trillion in 2030 (global GDP 2.2%). Therefore, policymakers need to take an urgent action to prepare health and social security systems to mitigate the effects of diabetes [2]. Diabetic foot ulcers are one of the most severe diabetes-related complications [3]. It is estimated that 19% to 34% of the diabetic population will develop diabetic foot ulcers in their lifetime [4]. Therefore, prevention of diabetic foot ulcers plays an essential role in the care of people with DM [5, 6]. Policymakers need to take an urgent action to prepare health and social security systems to mitigate the effects of diabetes [7].

Walking is the most common physical activity in activities of daily living [8]. There is sufficient evidence to support that walking is an effective intervention for people with DM. Walking can reduce postprandial glucose, insulin, and non-esterified fatty acid response compared to prolonged sitting in people with DM [9]. Various intensities of walking training have been used to improve health-related quality of life in people with DM [10]. Walking is the most common physical activity in activities of daily living [11]. Walking can reduce postprandial glucose, insulin, and non-esterified fatty acid response compared to prolonged sitting in people with DM [12]. Moreover, recent studies have shown that even short bouts of walking can ameliorate glucose profiles in diabetic patients with sedentary behavior [13, 14]. However, walking for people with DM may increase the risk of developing plantar skin breakdown by repetitive high vertical or shear stresses on the foot [15–17]. Up to date, the influences of various walking intensities on plantar tissue remain largely unknown in both healthy people and people with diabetes [17].

Peak plantar pressure (PPP) has been commonly used to predict the risk of diabetic foot ulcers [18–20]. However, Lavery et al. indicated that the PPP alone is not an adequate diagnostic tool to identify high-risk diabetic foot ulcers [21]. Mueller et al. introduced another useful indicator, peak pressure gradient (PPG), for characterizing the spatial change in plantar pressure across adjacent sites of the foot surface around the PPP [22]. PPG provides information concerning plantar pressure distribution and the damaging internal stresses within the foot soft tissues. PPG may contribute to skin breakdown because PPG may result in shear stresses within the soft tissues [22]. Therefore, PPG may be more discriminating than PPP alone for developing a foot ulcer [23–27].

PPG is calculated based on pressure distributions during the overall contact time without considering time-varying features of pressure notes during the gait cycle [28]. The directions of consecutive maximal pressure gradients may vary during the stance phase of the gait cycle [27]. Therefore, the gradient direction of the variation, defined as the pressure gradient angle (PGA) in this study, may cause a more complex deformation of foot soft tissues, even if PPG magnitude and location remain the same. PGA provides additional information to quantify the time-varying directional angle of instantaneous PPG. Additionally, increased PGA decreases the pressure concentration, and the value of PGA can offer a new window to study the influence of plantar pressures on foot soft tissue [27]. With advanced understanding of the effect of dynamic plantar pressures during various intensities of walking could shed light on the plantar tissue deformation and stress.

Supriadi et al. argued that there would be a cut-off value of pressure gradient for the risk threshold of foot ulcers [29]. Therefore, quantifying the walking intensity, including different speeds and durations and their effect on PPG and PGA in people with DM, is essential for prescribing suitable walking exercise and rehabilitation interventions. However, to the best of our knowledge, there is no study investigating the effect of various intensities of walking exercise, including different speeds and durations, on PPG and PGA values of the plantar foot in people without and with DM. Thus, it is essential to study the response of PPG and PGA to different walking speeds and durations in healthy people first. The results can provide a foundation to understand the effect of diabetes on PPG and PGA patterns to various weight-bearing activities. Therefore, the current study aimed to examine the effect of different walking speeds and durations on PPG and PGA patterns in non-diabetics.

The purpose of this study were to propose a new index, pressure gradient angle, to quantify and characterize dynamic plantar pressure patterns during walking at various intensities and to investigate the effect of various walking speeds and durations on the plantar pressure gradient and pressure gradient angle.

## Methods

A 3 × 2 factorial design, including three walking speeds (1.8, 3.6, and 5.4 mph) and two durations (10 and 20 min), was used in this study. This was part of a larger project investigating plantar tissue in response to various walking intensities [8, 30].

### Subjects

Healthy subjects between 18 and 45 years of age were recruited from the university and nearby community. The inclusion criteria for this study were without any diagnosed diseases nor musculoskeletal pain of the lower extremity. The examinations were performed in the Rehabilitation Engineering Laboratory of the University of Illinois at Urbana-Champaign. Each subject signed the informed consent approved by the University of Illinois at Urbana-Champaign Institutional Review Board (#19,225) before the screening and experimental procedures [8, 30]. Twelve healthy participants (5 men and 7 women) were recruited in this study. The demographic data were: age, 27.1 ± 5.8 years; height, 1.7 ± 0.1 m; and weight, 63.5 ± 13.5 kg (mean ± standard deviation). The dominating leg of all subjects is the right side.

### Plantar pressure measurements

Participants performed all examinations at room temperature maintained at 24 ± 2 °C. All subjects relaxed in the supine position for at least 20 min before the walking protocol to avoid the influence of previous weight-bearing activities (e.g., walking to the lab) on the plantar pressures.

Participants wore a suitable pair of shoes and socks (Altrex, Teaneck, NJ, USA). Then F-scan in-shoe sensor (Tekscan, South Boston, MA) with a sampling rate of 300 Hz was placed between the sock and the insole to measure the plantar pressure of the right foot [27]. An F-scan in-shoe sensor contains 960 sensing elements. The size of each sensing element is 5.08 mm × 5.08 mm. The subjects were permitted multiple practice trials (5 trials on the average per subject) to acclimate to the insole pressure system and the treadmill. A total of 6 walking protocols was tested in this study. The participant received the 1.8 mph protocol in the first week, the 3.6 mph protocol in the second week, and the 5.4 mph protocol in the third week. The order of duration (10 and 20 min) was randomly assigned [8, 30]. Each protocol was separated by 7 ± 2 days.

### Data analysis

The plantar pressure data were analyzed in the average values of the three intermediate steps from the last minute of each trial. The four regions at high risk of foot ulcers were selected for this study and included the first toe (T1), first metatarsal head (M1), second metatarsal head (M2), and heel (HL) [31]. Plantar areas at low risk for foot ulcers were not selected in this study.

**Fig. 1:**
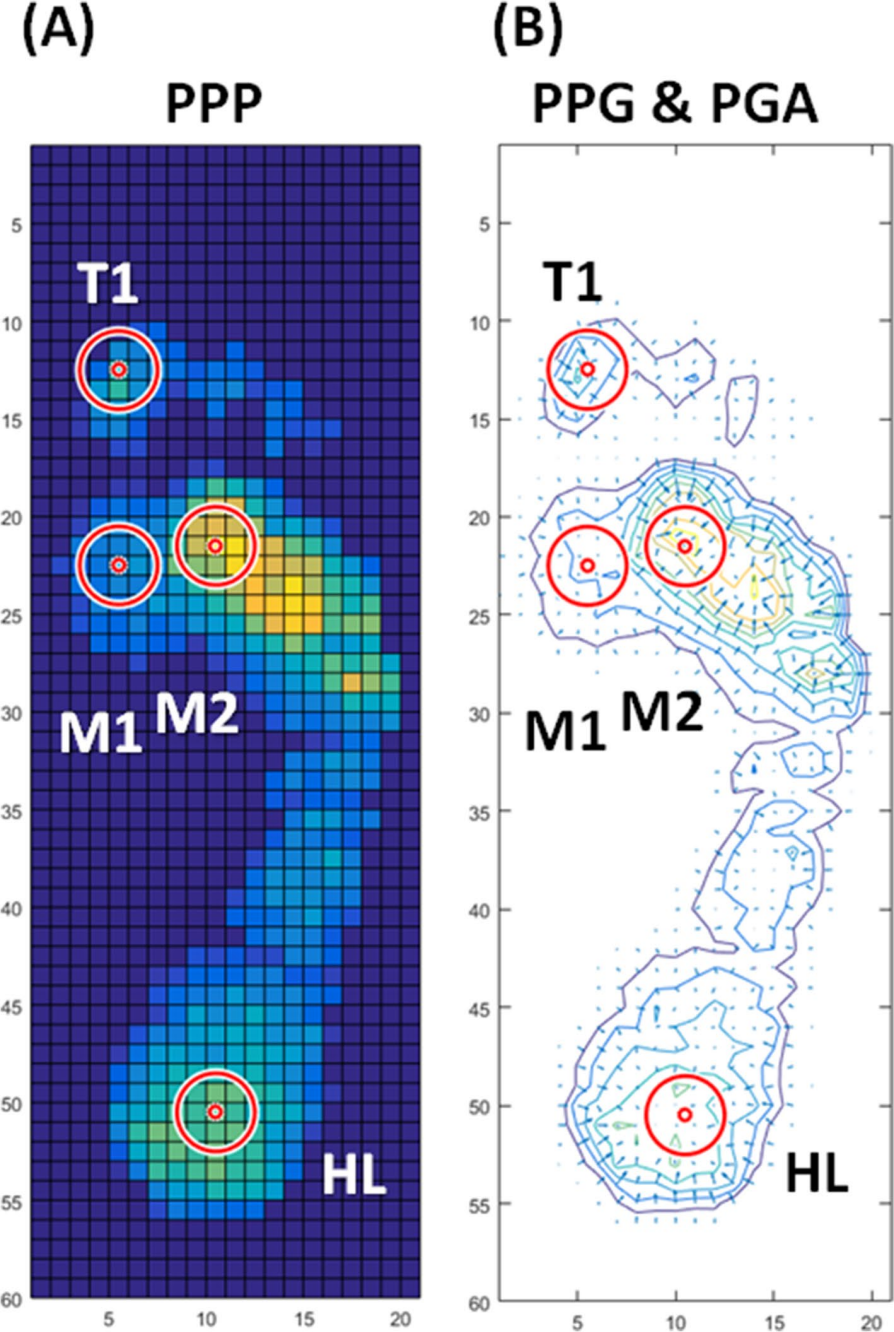
Examples of PPP (**A**) and PPG (**B**) in a representative participator at four plantar regions are defined. PPP, peak plantar pressure; PPG, peak pressure gradient; T1, first toe; M1, first metatarsal head; M2, second metatarsal head; and HL, heel

**Fig. 2:**
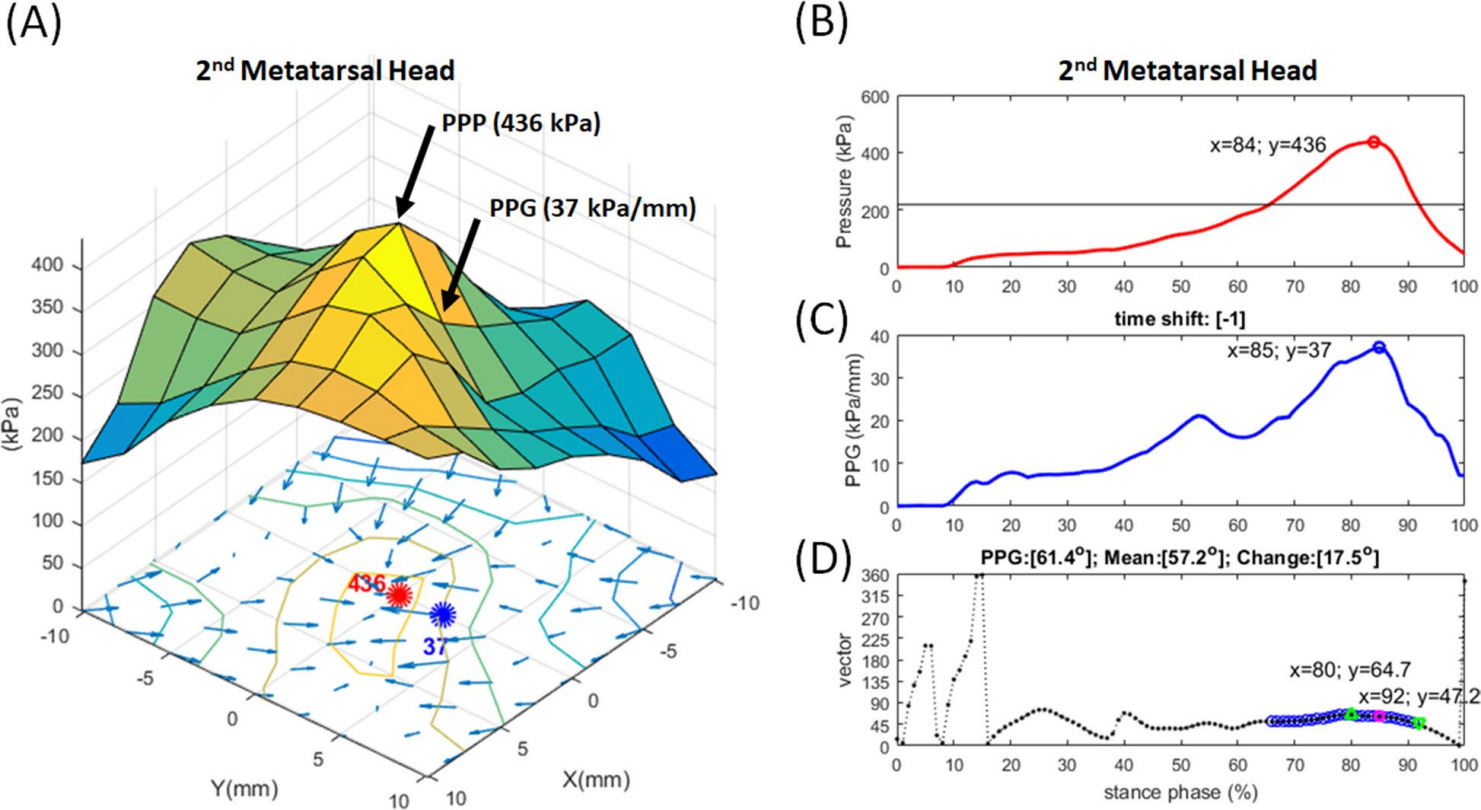
Examples of PPP, PPG, and PGA at the 2^nd^ metatarsal head in a representative participate. **A** PPP = 436 kPa and PPG = 37 kPa/mm. The PPP and PPG were not at the same point. **B** PPP was during the stance phase of gait. **C** PPG was during the stance phase of gait. **D** PGA was the angle change of the pressure gradient vector, which was instantaneous PPP more than half of the overall PPP. PGA = 17.5°. PPP, peak plantar pressure; PPG, peak pressure gradient; PGA, pressure gradient angle

The PPP was determined from the highest pressure in a defined area (5 × 5 F-Scan sensor pixels [645.2 mm2]). Furthermore, adding nodes between the sensor pixels was to increase the accuracy of pressure gradient calculation (Figs. 1A and 2A) [22]. A bicubic polynomial spline smoothing function was applied to the raw data of plantar pressures to eliminate individual pixel outliers and estimate pressure values at nodes located half the length between each sensor pixel. The PPP was calculated during a stance phase of the gait cycle (Figs. 1B and 2B) by the Eq. (1) [27]:

1 $$PPP= max (p)$$

where *p* is the plantar pressure distribution within each of the four plantar regions.

The gradient of *p* is defined as the unique vector field in the two-dimensional Cartesian coordinate system with a Euclidean metric. The PPG was determined at the highest gradient of *p* during a stance phase of the gait cycle (Fig. 2C). Finally, the PPG was calculated by the Eq. (2) [32]:

2 $$PPG=max(\nabla p)=max [{g}_{x},{g}_{y}] =max(\frac{\partial p}{\partial x}\genfrac{}{}{0pt}{}{\to }{i},\frac{\partial p}{\partial y}\genfrac{}{}{0pt}{}{\to }{j})$$

where *i* and *j* are the standard unit vectors in the directions of the *x* and *y* coordinates, respectively, ${g}_{x}$ is a gradient in the *x*-direction, ${g}_{y}$ is a gradient in the *y*-direction, $\frac{\partial p}{\partial x}$ is the partial derivative for x, $\frac{\partial p}{\partial y}$ is the partial derivative for *y*, and *∇p* is the pressure gradient.

The pressure gradient magnitudes were calculated by subtracting the pressure in the adjacent node of the *p-*note, then dividing by the distance between the nodes. Thus, the formula calculates the pressure gradient magnitude:

3 $$\nabla p=\sqrt{{({g}_{x})}^{2}+{({g}_{y})}^{2}}$$

The gradient direction *θ* can be determined by considering the directional variations of the peak gradient vector. The gradient direction *θ* can be computed from the dot product of the magnitudes of the two vectors (${g}_{y}$ and ${g}_{x}$). Thus, the gradient direction *θ* is defined as:

4 $$\theta ={tan}^{-1}[\frac{{g}_{y}}{{g}_{x}}]$$

PGA can be determined by considering the directional variations of the peak gradient vector. PGA defines the range between the maximal and minimal gradient direction *θ* during a stance phase of the gait cycle (Fig. 2D). Thus, the equation of PGA [27]) is defined as:

5 $$PGA={Max}_{1\le i\le N} ({\theta }_{i})-{Min}_{1\le i\le N} ({\theta }_{i})$$

where *θ* is the gradient direction of the pressure gradient vector at the *i*-th time index, and *N* is the time index when the instantaneous PPP is more than half of the overall PPP. As shown in our previous study, the results of PGA were stable when the PGA was calculated by the instantaneous PPP of more than 50% of sensors. Therefore, the selection of pressures with more than half PPP is to exclude unstable PGA associated with small plantar pressures.

### Statistical analysis

The PPP, PPG, and PGA values were presented as the mean ± standard error. A 3 × 2 two-way analysis of variance (ANOVA) with repeated measures was used to compare the PPG and PGA values among the three speeds (slow walking 1.8, brisk walking 3.6, and slow running 5.4 mph) and two durations (10 and 20 min) and the interaction between the speeds and durations [8, 30]. The two-way ANOVA was used to examine the effect of two main factors (the speed factor and the duration factor) on PPG and PGA and the interaction between the speed and duration factors on PPG and PGA [8, 30]. A one-way ANOVA with Fisher’s LSD post hoc test was used for pairwise comparisons of the PPG and PGA between three walking speeds (1.8, 3.6, and 5.4 mph) under each walking durations (10 and 20 min). The differences in the PPG and PGA between two walking durations (10 and 20 min) under each walking speed (1.8, 3.6, and 5.4 mph) were examined using the Student’s *t*-test. Furthermore, correlations between PPP, PPG, and PGA were determined using a Pearson product-moment correlation analysis. A significance level of 0.05 was used for all analyses.

## Results

**Fig. 3:**
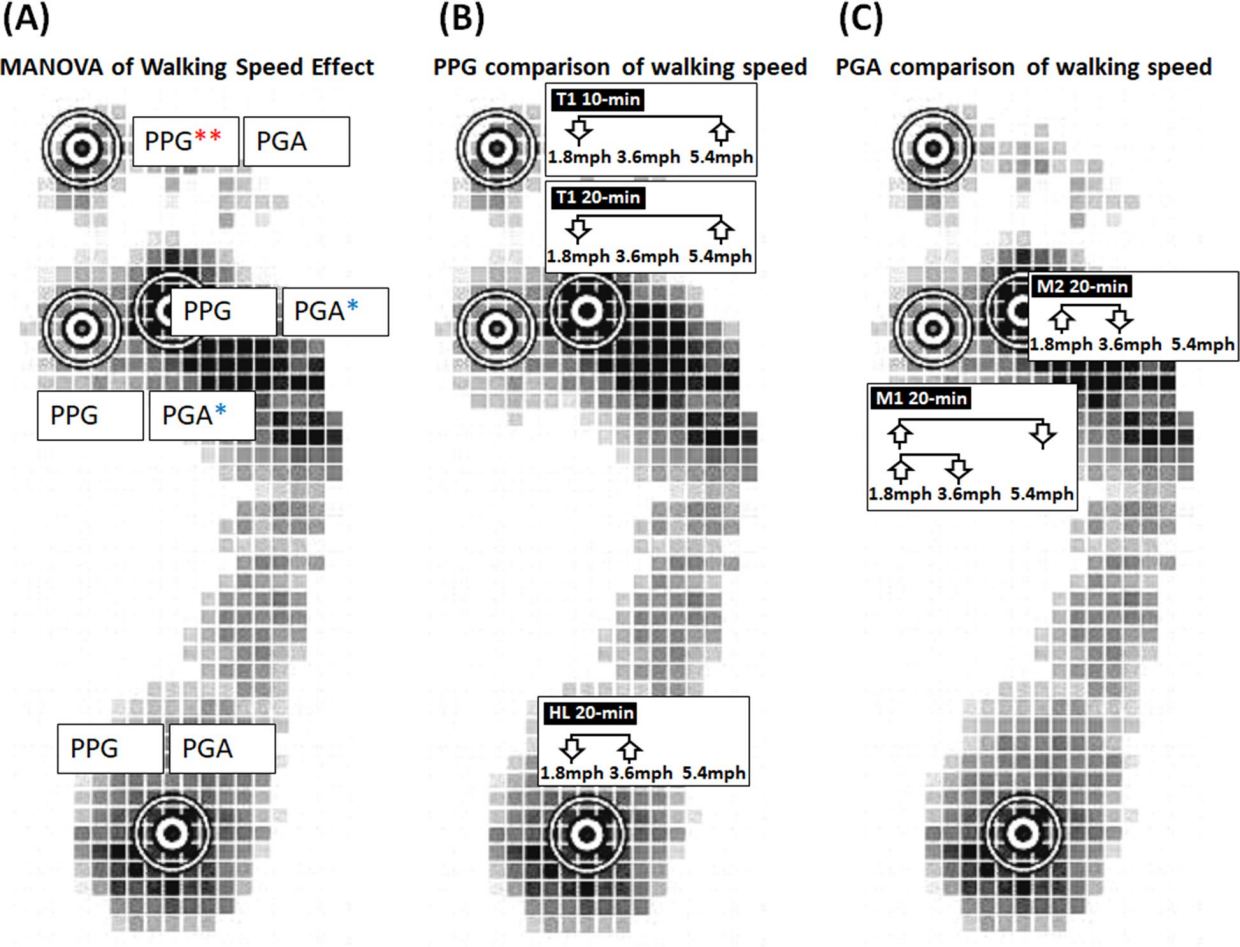
Illustration of the effect of walking speeds on the PPG and PGA. **A** 3 × 2 two-way ANOVA (3 speeds and 2 durations) showed that the speed factor caused a significant main effect of PPG in T1. **B** PPG in one-way ANOVA showed 1.8 mph were lower than 5.4 mph in T1 at 10 min and 20 min, and 1.8 mph were lower than 3.6 mph in HL at 20 min. **C** PGA in the one-way ANOVA showed 1.8 mph were higher than 3.6 and 5.4 mph in M1 at 20 min, and 1.8 mph were higher than 3.6 mph in M2 at 20 min. PPG, peak pressure gradient; PGA, pressure gradient angle; T1, first toe; M1, first metatarsal head; M2, second metatarsal head; and HL, heel

In the interaction between the speed and duration on PPG and PGA, the 3 × 2 two-way ANOVA (3 speeds and 2 durations) showed that the speed factor caused a significant main effect of PPG in T1 (*p* = 0.008), and PGA in both of M1 (*p* = 0.012) and M2 (*p* = 0.037). However, the duration factor did not significantly change the PPG and PGA. There was no interaction between the speed and duration factors on PPG and PGA (Fig. 3A).

**Fig. 4:**
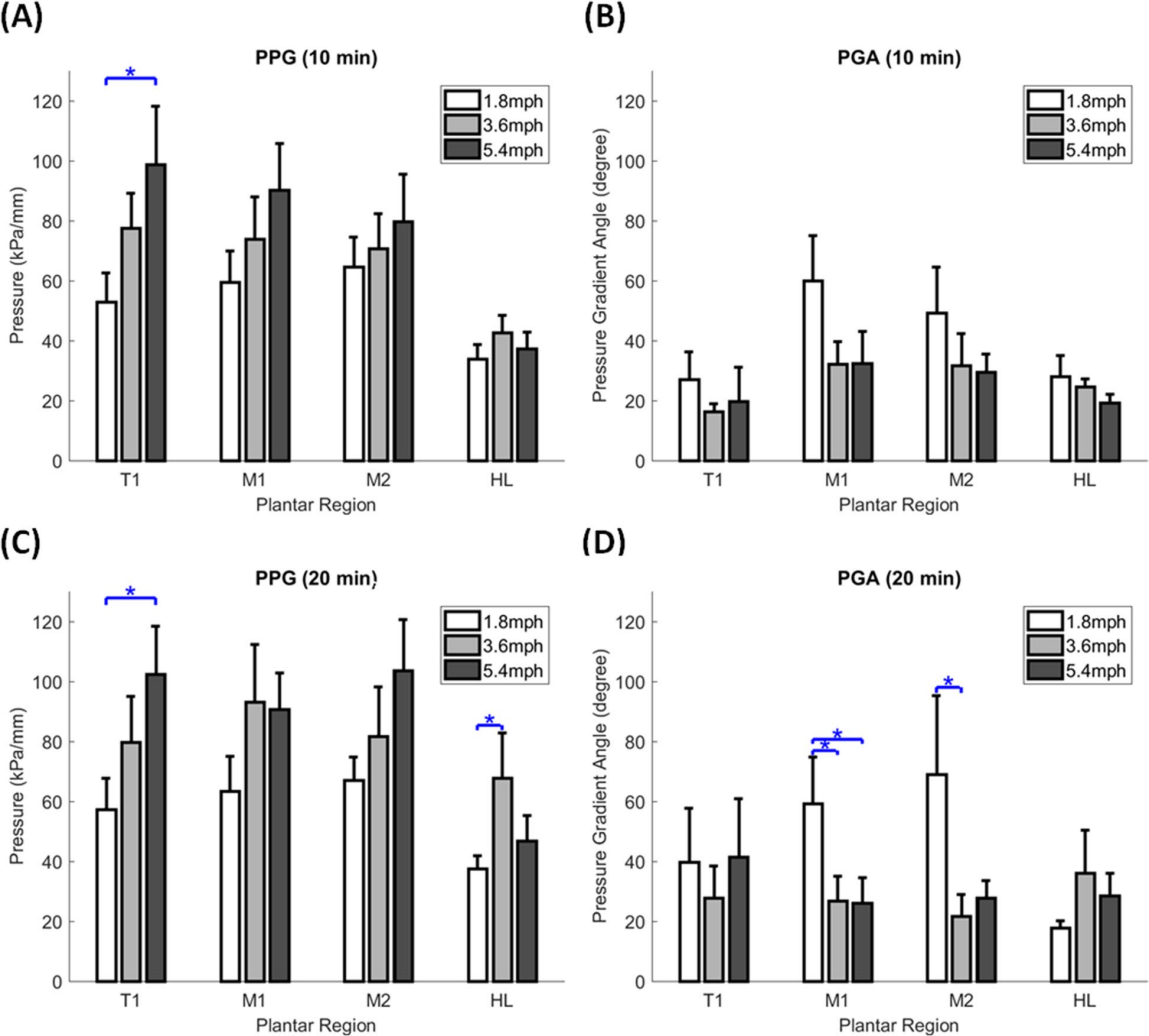
Comparisons of the effect of walking speeds on the PPG and PGA of the four plantar regions at two walking durations. **A** PPG at 10 min walking duration. **B** PPG at 20 min walking duration. **C** PGA at 10 min walking duration. **D** PGA at 20 min walking duration. Data are shown as mean ± standard errors. *, a significant difference (*p* < 0.05). PPG, peak pressure gradient; PGA, pressure gradient angle; T1, first toe; M1, first metatarsal head; M2, second metatarsal head; and HL, heel

In the effect of walking speeds on PPG, the one-way ANOVA showed that walking speed of 1.8 mph were lower than other speeds in three significant differences: (1) 10 min in T1, between 1.8 and 5.4 mph (53.0 ± 9.6 vs. 98.7 ± 19.4 kPa/mm, *p* = 0.029); (2) 20 min in T1 between walking speed of 1.8 and 5.4 mph (57.4 ± 10.4 vs. 102.4 ± 16.0 kPa/mm, *p* = 0.031); and (3) 20 min in HL between 1.8 and 3.6 mph (37.5 ± 4.4 v.s. 67.8 ± 15.1 kPa/mm, *p* = 0.046) (Table 1, Fig. 3B, Fig. 4A and B).

**Table 1:** Effect of walking speeds on the PPG and PGA

Parameter	Duration	Region	Speed									One-way	Fisher’s LSD				
												**ANOVA**	**Post hoc**				
			1.8 mph (Mean ± SE)			3.6 mph (Mean ± SE)			5.4 mph (Mean ± SE)			*p*-value	1.8 mph Vs 3.6 mph		1.8 mph Vs 5.4 mph		3.6 mph vs 5.4 mph
**PPG**	10 min	T1	**53.0**	** ± **	**9.6**	77.6	±	11.6	**98.7**	** ± **	**19.4**	0.088	0.227		**0.029**	*	0.298
**(kPa/mm)**		M1	59.6	±	10.2	73.9	±	14.0	90.3	±	15.5	0.284	0.456		0.116		0.395
		M2	64.7	±	9.8	70.7	±	11.7	79.8	±	15.7	0.699	0.740		0.404		0.613
		HL	33.9	±	4.7	42.6	±	5.9	37.4	±	5.4	0.522	0.260		0.646		0.501
	20 min	T1	**57.4**	** ± **	**10.4**	79.8	±	15.2	**102.4**	** ± **	**16.0**	0.093	0.270		**0.031**	*	0.265
		M1	63.5	±	11.4	93.2	±	19.1	90.7	±	12.2	0.294	0.160		0.196		0.907
		M2	67.0	±	7.7	81.8	±	16.4	103.7	±	17.0	0.206	0.469		0.079		0.289
		HL	**37.5**	** ± **	**4.4**	**67.8**	** ± **	**15.1**	46.7	±	8.6	0.120	**0.046**	*	0.531		0.158
**PGA**	10 min	T1	27.0	±	9.2	16.4	±	2.6	19.8	±	11.4	0.677	0.391		0.557		0.784
**(degree)**		M1	60.0	±	15.0	32.3	±	7.3	32.4	±	10.7	0.158	0.096		0.097		0.996
		M2	49.3	±	15.2	31.8	±	10.4	29.6	±	5.8	0.403	0.275		0.221		0.892
		HL	28.1	±	7.0	24.6	±	2.5	19.3	±	2.8	0.409	0.593		0.187		0.425
	20 min	T1	39.8	±	17.9	27.8	±	10.5	41.5	±	19.4	0.815	0.610		0.941		0.560
		M1	**59.2**	** ± **	**15.5**	**26.8**	** ± **	**8.1**	**26.1**	** ± **	**8.4**	0.073	**0.050**	*	**0.045**	*	0.963
		M2	**69.0**	** ± **	**26.2**	**21.7**	** ± **	**7.2**	27.9	±	5.5	0.091	**0.044**	*	0.078		0.784
		HL	17.7	±	2.3	36.2	±	14.2	28.6	±	7.4	0.384	0.172		0.416		0.570

*PPG* Peak pressure gradient, *PGA* Pressure gradient angle, *T1* First toe, *M1* First metatarsal head, M2 Second metatarsal head, and *HL* Heel; Data are shown as mean ± standard errors

^*^, a significant difference (*p* < 0.05)

In the effect of walking speeds on PGA, the one-way ANOVA showed that walking speed of 1.8 mph was greater than other speeds at walking duration 20 min in three significant differences: (1) M1 between 1.8 and 3.6 mph (59.2 ± 15.5 vs. 26.8 ± 8.1 degree, *p* = 0.050); (2) M1 between 1.8 and 5.4 mph (59.2 ± 15.5 vs. 26.1 ± 8.4 degree, *p* = 0.045); and (3) M2 between 1.8 and 3.6 mph (69.0 ± 26.2 vs. 21.7 ± 7.2 degree, *p* = 0.044) (Table 1, Fig. 3C, Fig. 4C and D).

**Fig. 5:**
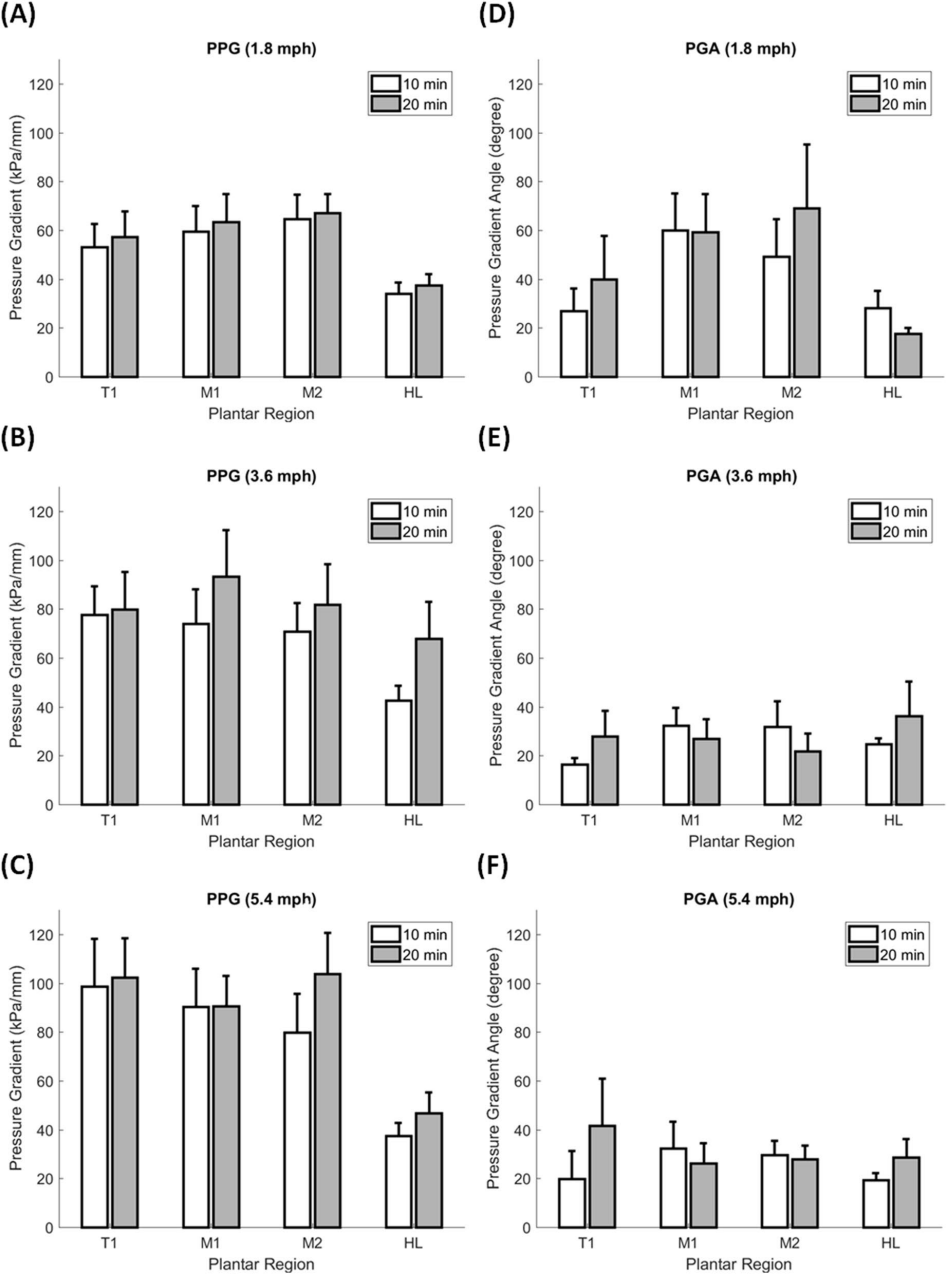
Comparisons of the effect of walking durations on the PPG and PGA of the four plantar regions at three walking durations. **A** PPG at 1.8 mph walking speed. **B** PPG at 3.6 mph walking speed. **C** PGA at 5.4 mph walking speed. **D** PGA at 1.8 mph walking speed. **E** PGA at 3.6 mph walking speed. **F** PGA at 5.4 mph walking speed. Data are shown as mean ± standard errors. PPG, peak pressure gradient; PGA, pressure gradient angle; T1, first toe; M1, first metatarsal head; M2, second metatarsal head; and HL, heel

In the effect of walking durations on PPG and PGA, there were no significant pairwise differences. However, the PPG has been trending lower in the 10 min compared with 20 min (Table 2 and Fig. 5).

**Table 2:** Effect of walking duration on the PPG and PGA

	Speed	Region	Duration						Paired *t*-test
			10 min (Mean ± SE)			20 min (Mean ± SE)			*p*-value
PPG	1.8 mph	T1	53.0	±	9.6	57.4	±	10.4	0.513
		M1	59.6	±	10.2	63.5	±	11.4	0.459
		M2	64.7	±	9.8	67.0	±	7.7	0.769
		HL	33.9	±	4.7	37.5	±	4.4	0.425
	3.6 mph	T1	77.6	±	11.6	79.8	±	15.2	0.841
		M1	73.9	±	14.0	93.2	±	19.1	0.116
		M2	70.7	±	11.7	81.8	±	16.4	0.539
		HL	42.6	±	5.9	67.8	±	15.1	0.082
	5.4 mph	T1	98.7	±	19.4	102.4	±	16.0	0.850
		M1	90.3	±	15.5	90.7	±	12.2	0.976
		M2	79.8	±	15.7	103.7	±	17.0	0.211
		HL	37.4	±	5.4	46.7	±	8.6	0.141
PGA	1.8 mph	T1	27.0	±	9.2	39.8	±	17.9	0.502
		M1	60.0	±	15.0	59.2	±	15.5	0.958
		M2	49.3	±	15.2	69.0	±	26.2	0.418
		HL	28.1	±	7.0	17.7	±	2.3	0.217
	3.6 mph	T1	16.4	±	2.6	27.8	±	10.5	0.244
		M1	32.3	±	7.3	26.8	±	8.1	0.571
		M2	31.8	±	10.4	21.7	±	7.2	0.415
		HL	24.6	±	2.5	36.2	±	14.2	0.451
	5.4 mph	T1	19.8	±	11.4	41.5	±	19.4	0.378
		M1	32.4	±	10.7	26.1	±	8.4	0.276
		M2	29.6	±	5.8	27.9	±	5.5	0.767
		HL	19.3	±	2.8	28.6	±	7.4	0.277

*PPG* Peak pressure gradient, *PGA* Pressure gradient angle, *T1* First toe, *M1* First metatarsal head, *M2* Second metatarsal head, and *HL* Heel; Data are shown as mean ± standard errors

**Fig. 6:**
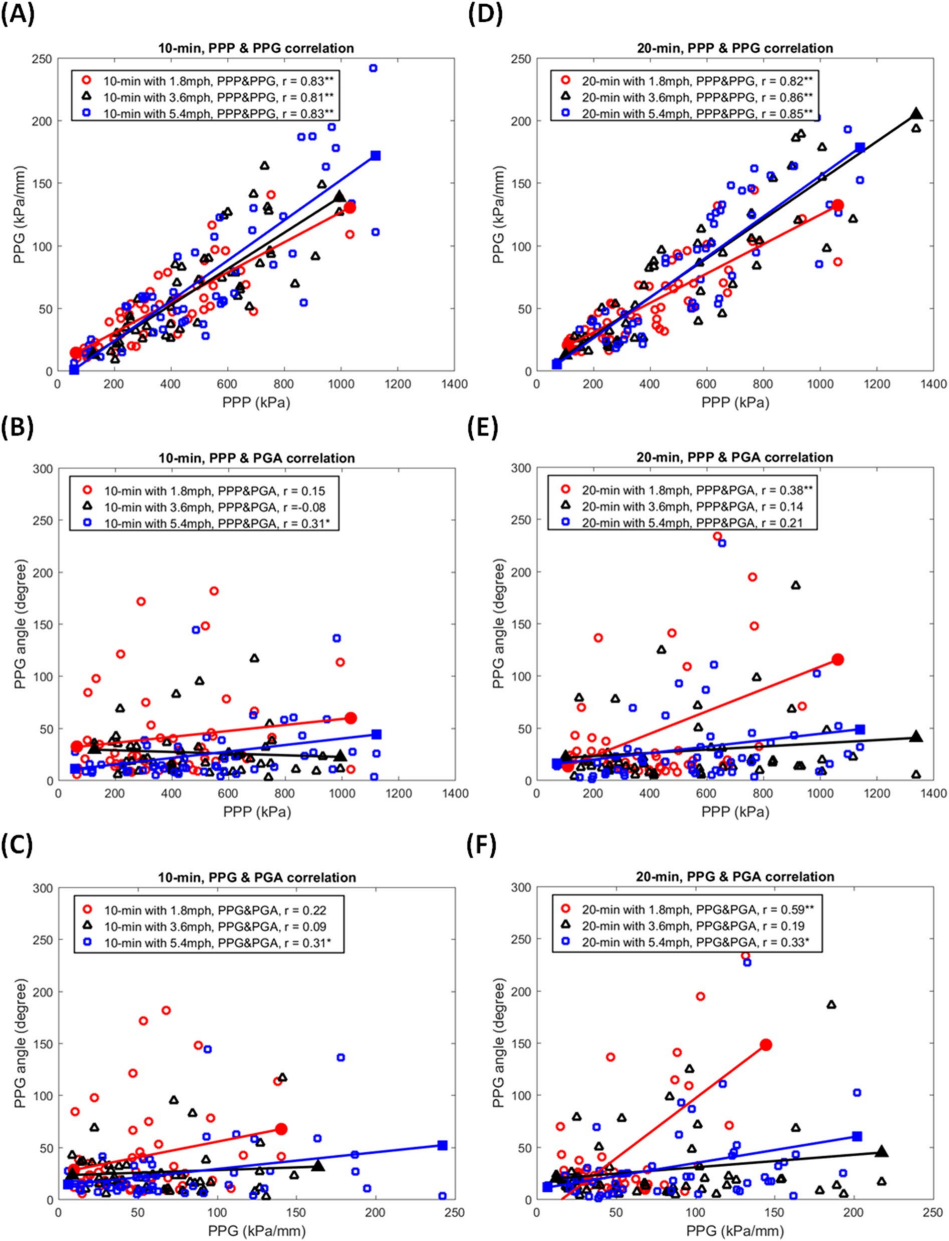
The scatter plots show the relationships among the PPP, PPG, and PGA in three walking durations at two walking durations. **A** PPP versus PPG at 10 min walking duration. **B** PPP versus PGA at 10 min walking duration. **C** PPG versus PGA at 10 min walking duration. **D** PPP versus PPG at 20 min walking duration. **E** PPP versus PGA at 20 min walking duration. **F** PPG versus PGA at 20 min walking duration. PPP, peak plantar pressure; PPG, peak pressure gradient; PGA, pressure gradient angle; T1, first toe; M1, first metatarsal head; M2, second metatarsal head; and HL, heel. *, a significant correlation (*p* < 0.05); **, a significant correlation (*p* < 0.01)

**Fig. 7:**
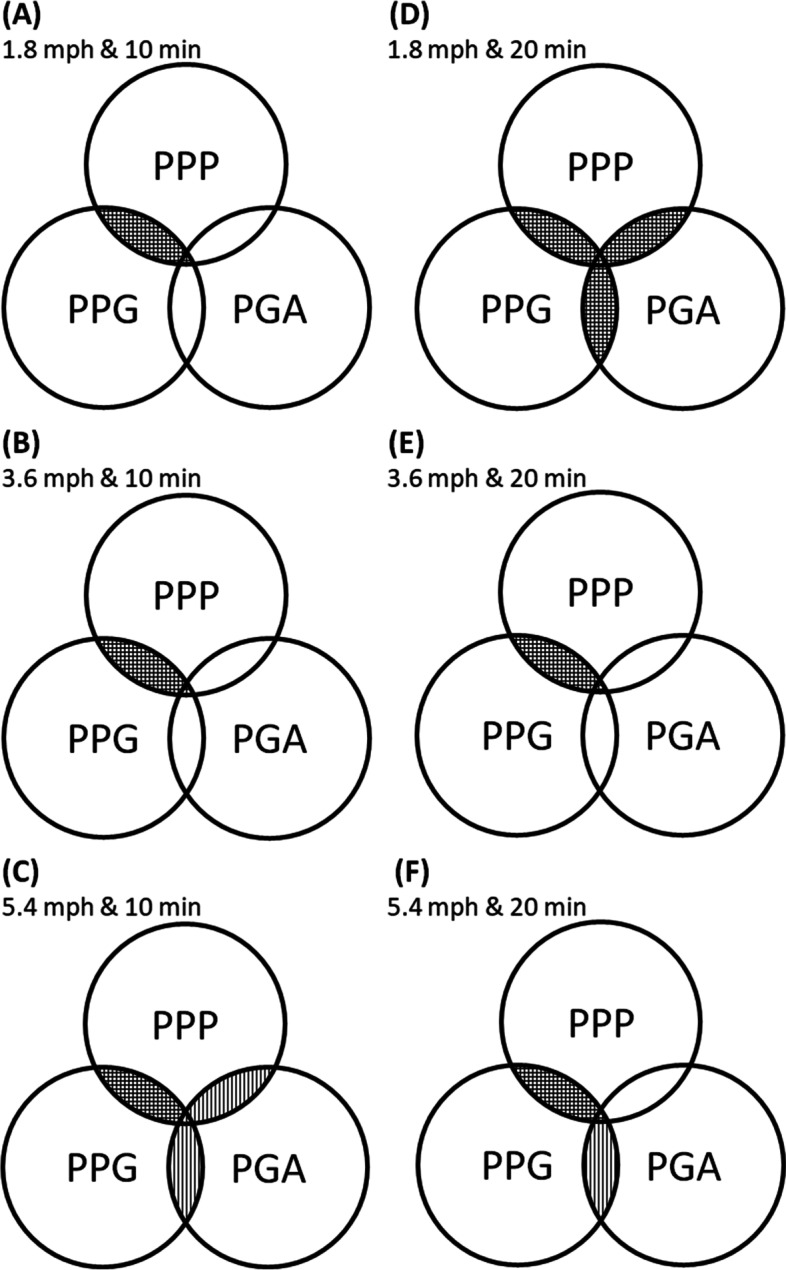
Illustration of relationships among the PPP, PPG, and PGA in three walking speeds and two walking duration. The Overlaps indicated a significant correlation. **A** 1.8 mph at 10 min. **B** 3.6 mph at 10 min. **C** 5.4 mph at 10 min. **D** 1.8 mph at 20 min. **E** 3.6 mph at 20 min. **F** 5.4 mph at 20 min. PPP, peak plantar pressure; PPG, peak pressure gradient; PGA, pressure gradient angle; **▥** parallel-line, a significant correlation (*p* < 0.05); **▦** cross-line, a significant correlation (*p* < 0.01)

In the correlation between the PPP, PPG, and PGA, the PPP has six significant correlations with PPG in 10 and 20 min walking duration with three walking speeds (*r* = 0.808 ~ 0.865, *p* < 0.001). Furthermore, there were another five significant correlations between PGA with PPP or PPG. In the first and second correlations, at 10 min walking duration with a walking speed at 5.4 mph, PGA has a significant correlation with PPP (*r* = 0.309, *p* = 0.032) and PPG (*r* = 0.308, *p* = 0.003). In the third and fourth correlations, at 20 min walking duration with a walking speed at 1.8 mph, PGA has a significant correlation with PPP (*r* = 0.383, *p* = 0.007) and PPG (*r* = 0.591, *p* < 0.001). Finally in the fifth correlation, at 20 min walking duration with a walking speed at 5.4 mph, PGA has a significant correlation with PPG (*r* = 0.332, *p* = 0.021) (Table 3, Fig. 6, and Fig. 7).

**Table 3:** Correlation coefficients among PPP, PPG, and PGA in three walking durations (1.8, 3.6, and 5.4 mph) at two walking durations (10 and 20 min)

		PPP and PPG			PPP and PGA			PPG and PGA		
Duration	Speed	*r*	*p*-value		*r*	*p*-value		*r*	*p*-value	
10 min	1.8 mph	0.831	< 0.000	**	0.147	0.319		0.223	0.127	
	3.6 mph	0.808	< 0.000	**	-0.078	0.599		0.089	0.546	
	5.4 mph	0.834	< 0.000	**	0.309	0.032	*	0.308	0.033	*
20 min	1.8 mph	0.819	< 0.000	**	0.383	0.007	**	0.591	< 0.001	**
	3.6 mph	0.865	< 0.000	**	0.136	0.356		0.193	0.189	
	5.4 mph	0.846	< 0.000	**	0.213	0.146		0.332	0.021	*

*PPG* Peak pressure gradient, *PGA* Pressure gradient angle, *T1* First toe, *M1* First metatarsal head, *M2* Second metatarsal head, and *HL* Heel; Data are shown as correlation coefficients

^*^, a significant difference (*p* < 0.05)

^**^, a significant difference (*p* < 0.01)

## Discussion

This study demonstrated that the walking speeds (1.8, 3.6, and 5.4 mph) significantly affected PPG and PGA. However, the walking durations (10 and 20 min) did not significantly affect PPG and PGA. Our results indicate that PPG in the first toe region after fast walking speed (5.4 mph) for either 10 or 20 min was significantly higher than slow walking speed (1.8 mph) (Fig. 3B). Meanwhile, PPG in the heel region after moderate walking speed (3.6 mph) for 20 min was significantly higher than slow walking speed (1.8 mph) (Fig. 3B). Results also indicate that PGA in the forefoot region after moderate walking speed (3.6 mph) for 20 min was significantly narrower compared to slow walking speed (1.8 mph) (Fig. 3C). Therefore, this study suggests that slow walking (1.8 mph) would be a cut-off value of PPG and PGA for the risk threshold of foot ulcers [29].

The higher PPG and narrower PGA associated with higher walking speed may be more discriminating than higher peak plantar pressure alone of individuals at risk of developing a foot ulcer [22, 27, 33]. Mueller and Maluf proposed the physical stress theory to provide an appropriate intensity of exercise that needed physical stress to maintain tissue health [34]. According to the physical stress theory, tissue injury may occur during unsuitable walking intensity. However, there are no definitive values of the appropriate walking intensity for various tissues. This study demonstrated that walking at slow walking speed resulted in lower PPG than other walking speeds. Our finding also showed that the wider PGA after slow walking speed might decrease the potential for skin injury [28]. These results implied that a slow walking speed at 1.8 mph might be an appropriate strategy for people to prevent the risk for foot ulcers.

This study demonstrated that PPG under the first toe was affected by the walking speed. Our results showed that higher PPG was found in the first toe at 5.4 mph compared to 1.8 mph. The results, same with our previous study, indicated that the higher PPG in the first toe might be a higher prevalence of foot ulcers [27]. In particular, the first toe constitutes one-third of all areas affected by diabetic foot ulcers [35]. In addition, first toe re-ulceration can lead to hallux amputation, which has devastating effects on foot biomechanics and increases the risk of new ulcers and lower-extremity amputation [36]. The higher PPG in the first toe during high walking speed may relate to the first metatarsophalangeal joint range motion. The dorsiflexion motion range is usually defined as more than 40° in the first metatarsophalangeal joint [37, 38]. Zhang et al. showed that the walking speed decreased significantly after the first metatarsophalangeal joint was restricted [39]. It indicated that the high walking speed might need more range of motion of the first metatarsophalangeal joint. Wu et al. demonstrated that increased flexion resulted in decreased compressive force during the walking, however, increased shear force [40]. These were consistent with the findings in this study. We speculate that the first toe during high walking speed has greater PPG contributing to skin breakdown because they generate significant shear stresses within the soft tissues [23].

This study also found that the PPG in the heel region was higher at moderate walking speed (3.6 mph) compared to slow walking speed (1.8 mph). The heel region is thicker and stiffer than other plantar regions [41]. Plantar pressure during walking is usually dissipated by the cushioning effects of the heel fat pad, a highly fibrous adipose structure [42]. The multiscale entropy algorithm observes that moderate walking speed has the highest complexity structure in stride interval time than slow and fast walking speed [43, 44]. Under periodic and most increased complexity foot pressure, the shear stress of the plantar soft tissue will increase in stimulating soft tissue failure [45], and the phenomenon could be termed fatigue [46]. These results indicate that moderate walking speed with the highest complexity structure of the plantar heel region in stride interval time may induce higher PPG.

This study showed that the PGA was narrower in moderate walking speed (3.6 mph) compared to slow walking speed (1.8 mph) in the medial forefoot (i.e., first and second metatarsal heads). The narrower PGA may relate with the plantar center of pressure (CoP) progression during the slow walking speed. The CoP progression is a path formed by a series of coordinates passing from the hindfoot through to the forefoot during the stance phase [47]. CoP trajectory time progress in the medial forefoot region is near terminal stance (60% to 90%) of walking stance time [48]. The giant CoP medial–lateral displacement is believed to be an adaptation strategy and the redistributed plantar pressure [49, 50], especially to the medial forefoot regions [49, 51]. As the walking speed increased, even the walking stance time decreased, the percent of walking stance time in CoP progression increased in the medial forefoot region for the push-off phase [52]. Our results showed that the moderate and fast walking speed (3.6 and 5.4 mph) might insufficient redistributed plantar pressure for the more push-off phase in the forefoot region. Furthermore, the forefoot region may appear the smaller CoP medial–lateral displacement to narrow the PGA in the forefoot.

The scatter plots of this study showed a significant correlation between PPP and PPG in each walking condition that was consistent with our previous study (Fig. 6A and D) [27, 33]. Our findings support that PPG is an adequate diagnostic tool to assist PPP in identifying high-risk diabetic foot ulcers [21]. In addition, this study showed that PGA was both a significant correlation with PPP and PPG in two walking intensities. One was at a short walking duration (10 min) with a fast walking speed (5.4 mph) (Fig. 7C). The other was at a long walking duration (20 min) with a slow walking speed (1.8 mph) (Fig. 7D). This result showed that the PGA might correlate with PPP and PPG during the suitable range of walking intensity. Schafer et al. found that repeat loading increased the soft tissue stiffness initially, however after a period of repeat loading, stiffness decreased [53]. In addition, after a period of repeat loading, the soft tissue skin blood perfusion can be affected by the specific accumulated mechanical stimulus [54]. Our results showed that PGA might simultaneously increase with PPP and PPG during this suitable range of accumulated mechanical stimulation during walking at various intensities. It is recommended that increased PPP and PPG during these walking intensities may induce a high risk of foot ulcers; at this present, increased PGA may play an essential role in the potential interventions for preventing foot ulcers.

Our findings have a potential impact on the assessment of foot ulcer risk. Traditional methods focus on maximal magnitude of planar pressure and ignore the dynamic changes of planar pressure patterns during various activities of daily living. Using six intensities of walking exercise, we demonstrated that walking intensities can cause different PPG and PGA patterns even under similar peak plantar pressure. Our proposed method on quantifying dynamic changes of plantar pressure patterns can be used to assess the impact of various types and intensities of exercise on plantar tissue viability in people at risk for foot ulcers.

There are limitations to this study. The first limitation is the lack of time integral magnitudes validation in PPG and PGA for this walking intensity study. Yavuz found that the local peak shear stress and shear-time integral were induced higher foot ulcer risk [55], indicating the need to know the effect of walking intensity in the time integral magnitudes of PPG and PGA in plantar regions. The second limitation is that the sample size was small in this study, which tends to impede the power of the statistical analysis. However, the goal of this study was to lend support to our hypothesis that the walking intensity affects the plantar pressure gradient (e.g., PPG and PGA).

## Conclusion

This study demonstrated that the walking speed (1.8, 3.6, and 5.4 mph in this study) significantly affected plantar pressure gradient and pressure gradient angle (dynamic directional changes of plantar pressure gradient); and the walking durations at 10 and 20 min did not significantly affect plantar pressure gradient and pressure gradient angle. Our results indicate that walking at 1.8 mph significantly lowered plantar pressure gradient and increased pressure gradient angle compared to fast walking speeds at 3.6 and 5.4 mph. In this study, we introduced the index of pressure gradient angle that can further quantify the dynamic patterns of plantar pressure gradient during walking and successfully demonstrated that walking at 1.8 mph effectively increased pressure gradient angle for avoiding pressure concentration over a small area of the planar foot, especially in the forefoot region. Our method and findings may contribute to understanding the role of plantar pressures in the development of foot ulcers.

## Data Availability

The datasets generated and analyzed during the current study are not publicly available due to ongoing secondary data analysis for publications, but are available from the corresponding author on reasonable request.
